# One-Stage Hybrid Repair of Multiple Degenerative Aneurysms

**DOI:** 10.1155/2012/432127

**Published:** 2012-09-23

**Authors:** George N. Kouvelos, Nektario K. Papa, Eleni M. Arnaoutoglou, Christina Bali, Miltiadis I. Matsagkas

**Affiliations:** ^1^Vascular Surgery Unit, Department of Surgery, Medical School, University of Ioannina, Ioannina University Campus, S. Niarchos Avenue, 45110 Ioannina, Greece; ^2^Department of Anaesthesiology, Medical School, University of Ioannina, Ioannina University Campus, S. Niarchos Avenue, 45110 Ioannina, Greece

## Abstract

The development of multiple aneurysms in different segments of the arterial tree requiring treatment is a challenge for the vascular surgeon as their management often demands more than one surgical procedure. We report a case of a 71-year-old male suffering from multiple aneurysms in four different segments of the arterial tree in combination with disabling claudication of his left leg. The patient was managed in a single session with a combination of classic open surgical and endovascular techniques in order to treat his aneurysms and revascularize his leg. This case illustrates the prospect to combine classic open surgical and endovascular techniques for the optimal management of multileveled arterial pathology. Combined therapy simplifies management and allows the one-stage treatment of these patients, while minimizing the overall operative risk.

## 1. Introduction

 The development of more than one aneurysm requiring treatment in different arterial segments may occur especially in older subjects and in the presence of atherosclerosis [1–5]. Treating these frail patients was always a challenge for the vascular surgeon, as especially in the past their management demanded multiple open surgical procedures. The combined use of endovascular and open techniques in the same surgical setting has gained popularity over time as vascular surgeons have acquired increasing experience with endovascular interventions. Hybrid procedures provide a surgical option to patients who had otherwise been deemed inoperable, by using as many minimally invasive strategies as possible. We report a case of a 71-year-old male suffering from multiple aneurysms in four different arterial segments in combination with disabling claudication of his left leg. The patient underwent successful hybrid repair in a single anesthesiologic session.

## 2. Case Report

**Figure 1:**
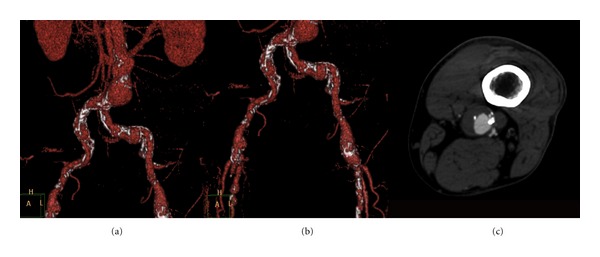
Three-dimensional reconstruction of computed tomography angiography illustrating the infrarenal abdominal aortic aneurysm (a) and aneurysms of both common femoral arteries (b). CT axial image revealing the aneurysm of the left popliteal artery (c).

 A 71-year-old non-Marfan male with a history of severe coronary artery disease, chronic obstructive pulmonary disease, and hyperlipidemia was referred to our department for evaluation of pulsatile masses in both groins and disabling (<20 m) intermittent claudication of his left limb. The CT angiogram revealed an infrarenal abdominal aortic aneurysm 5 cm in diameter, bilateral common femoral artery aneurysms (CFAAs) 2.8 cm (left) and 2.3 cm (right) in diameter, a thrombosed aneurysm of the right popliteal artery (PAA), and an aneurysm of the left popliteal artery 2.9 cm in diameter. Additionally diffuse stenotic lesions of the left SFA were noted (Figure 1). Distal runoff was adequate without any significant stenosis. After we took into consideration all the potential treatment options and given the high risk status of the patient, we decided to repair all four aneurysms and revascularize the left limb in a single session using a hybrid approach. The anatomic configuration of AAA fulfilled the requirements for EVAR. Based on this approach, under general anesthesia, the distal external iliac artery, the common femoral artery, and its bifurcation were exposed bilaterally through vertical incisions. Through direct puncturing of CFA's aneurysms, an endovascular bifurcated graft of 23 mm in main body diameter, 20 mm in limb diameter, and 14 cm in length was implanted (Excluder, W. L. Gore & Associates, Inc, Flagstaff, AZ, USA). Balloon remodeling was performed to the proximal and distal landing zones as well as to all overlapping sites according to the manufacturer's recommendations. Completion angiography revealed adequate graft deployment with exclusion of the aneurysm sac, maintenance of internal iliac arteries' perfusion, and no signs of endoleak. After removing the sheath from the right CFA to restore the flow in the right limb, we proceeded with open repair of the left CFA's aneurysm combined with the revascularization of the left leg. Initially resection of the CFA aneurysm was accomplished in combination with completion of the proximal anastomosis of a PTFE graft 6 mm in diameter. Afterwards, under direct catheterization and insertion of an 8F sheath, we proceeded with the deployment of a covered stent 7 mm in diameter and 10 cm in length (Viabahn, W. L. Gore & Associates, Inc, Flagstaff, AZ, USA) for the exclusion of the aneurysm of the left popliteal artery. Then, we proceeded with angioplasty and stenting of the stenoses of the SFA using two self-expandable covered stents 8 mm in diameter and 10 cm in length (Viabahn, W. L. Gore & Associates, Inc, Flagstaff, AZ, USA). At last we completed the distal anastomosis of the PTFE graft and restored the flow of the left leg. Finally, we proceeded in the resection of the right CFA aneurysm and accomplished the interposition of a 6 mm PTFE graft with restoration of the flow to the profunda femoral artery. The whole duration of the operation was 210 min, the amount of contrast media given was 320 mL (Optiray 320, Mallinckrodt Inc, St. Louis, USA), while the radiation burden was 72.1 mGy.

**Figure 2:**
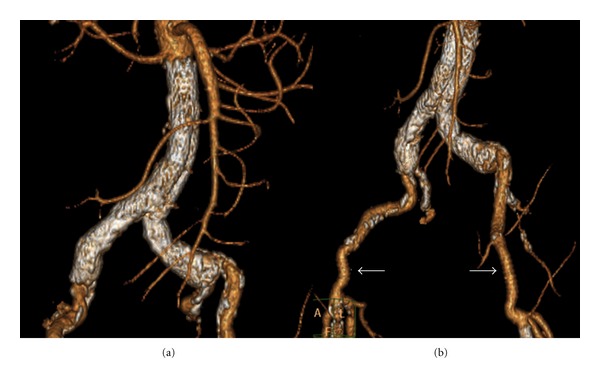
Six-month postprocedural computed tomography angiography revealing (a) the successful deployment of the aortic graft and (b) the patent femoral-femoral PTFE grafts (white arrows).

**Figure 3:**
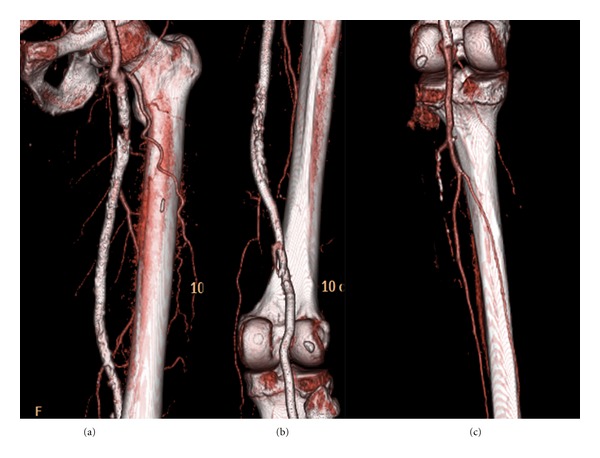
Three-dimensional reconstruction of computed tomography angiography demonstrating (a) the patent LSFA and (b) the exclusion of the sac of the aneurysm of the left popliteal artery with adequate distal runoff (c).

The patient experienced an uneventful recovery and was discharged at the 3rd postop day. At 6-month followup, the CT angiogram revealed no signs of endoleak and the patient was well having a normal life with no symptoms of intermittent claudication in his left leg anymore (Figures 2 and 3).

## 3. Discussion

 There is a strong association between the presence of true aneurysms involving the femoral or popliteal arteries and the presence of aneurysmal disease on other arterial segments [1]. It is well known that CFA aneurysms are associated with a high incidence of aneurysmal multiplicity of up to 69%–81% [2, 3]. There is also a clear association between popliteal and abdominal aortic aneurysms (AAAs), while the prevalence of popliteal aneurysms in patients with known AAA ranges between 3.2 and 14% [4, 5]. Multiple aneurysms show a male predominance, which is related to genetic and biologic factors that might be distinct from those contributing to patients with only AAA disease [5]. These patients may have some underlying connective tissue disorder as a predisposing genetic factor joint by hyperlipidemia and smoking [5]. In this setting, patients with peripheral aneurysms should be thoroughly investigated for other associated aneurysms, especially in cases of simultaneous atherosclerosis.

 Femoral artery aneurysms can reach a large size and may lead to leg swelling or pain from compression of the neighboring femoral vein and nerve. Occasionally, they could be limb threatening in case of thrombosis or as a source of embolic material. However, rupture is rarely encountered [6, 7].

The operative strategy of a femoral aneurysm depends on the involvement of the superficial or profunda femoral artery as well as on the patency of the femoropopliteal segment [8]. Few complications can be expected postoperatively [9]. Because of their rarity, no randomized trials have been performed comparing different techniques of repair. Usually, as in our patient, the caliber of the long saphenous vein is inadequate. Reversed saphenous vein graft should be used for mycotic or infected aneurysms [8, 10]. For localized aneurysms limited to the CFA, a short interposition graft is usually adequate and may sometimes serve as the origin of a femoropopliteal bypass graft [7]. No differences have been noted in terms of graft complications and thrombosis between ePTFE and Dacron grafts [8]. In our case, the presence of CFA aneurysms made somewhat problematic the access for the endovascular repair of AAA. We proceeded with direct puncturing of the CFA aneurysms in order to perform EVAR. This strategy facilitated the procedure, while the duration of ischemia of the right limb was significantly reduced. The flow to the right limb was restored immediately after the deployment of the aortic graft and remained so during the repair of the left leg.

According to the preference of the hybrid approach, the combination of open and endovascular techniques may allow treating these patients in a single anesthesiologic session. This strategy may reduce the possibility of addictive perioperative cardiac risk and may lead to avoidance of multiple surgical procedures, prolonged hospitalization, and surgical delays. Treating the patient for the limb ischemia and the popliteal aneurysm in another stage would probably have required puncturing, or, in case of open procedure, reoperating, on the graft of the CFA. This would have possibly jeopardized the graft's patency and also raised the risk of infection. Though a total open multistaged surgical procedure would have been an option in a fit and young patient, in our case the high-risk status of our patient led us follow a less invasive strategy.

 Although the indications for repair of PAAs are not well defined, a diameter greater than 2 cm to 3 cm, especially for aneurysms with a significant thrombus load or with chronic distal tibial artery embolic occlusion, is an acceptable indication for intervention [11]. Nevertheless, the optimal treatment approach remains a matter of debate. Johnson et al. in a retrospective analysis of 583 open operations for PAA reported a limb salvage rate of 99%, 97.6%, and 96.2% at 30 days, 1 year, and 2 years, respectively [12]. On the other side, Idelchik et al. reported that stent-graft exclusion of PAAs is safe and effective, yielding primary and secondary patency rates comparable to surgical repair (84.8% and 96.8%, resp.) [13]. Lovegrove et al. in a recent meta-analysis reported that endovascular repair of PAAs offers similar medium-term benefits but higher rates of early graft thrombosis and early interventions compared to open repair [14]. It is clear that although different case series [15, 16] and reports suggest the utility and technical success of endoluminal approach, long-term patency rates of endovascular repair of PAAs are still awaited. In our patient, there was an adequate landing zone for the graft in the PA, while simultaneously distal runoff was uncompromised. Based on these facts, we decided to proceed with the endovascular approach in treating the left PAA, in order to avoid the prolongation of the total operation time.

 In conclusion, this case illustrates the prospect to combine classic open surgical and endovascular techniques for the optimal management of multilevel arterial pathology. Combined therapy simplifies and allows the one-stage treatment of these patients leading to the reduction of the overall risk. In the endovascular era, single-step hybrid operations might overpass the need for multiple open procedures for the treatment of lesions in different vascular beds, resulting in improvement of patient's outcome.
